# The utility of cardiovascular magnetic resonance in the investigation of aborted sudden cardiac death

**DOI:** 10.1186/1532-429X-16-S1-O31

**Published:** 2014-01-16

**Authors:** Peter P Swoboda, Ananth Kidambi, Akhlaque Uddin, David P Ripley, Adam K McDiarmid, John P Greenwood, Sven Plein

**Affiliations:** 1 Multidisciplinary Cardiovascular Research Centre & Leeds Institute of Genetics, Health and Therapeutics, University of Leeds, Leeds, UK

## Background

Aborted sudden cardiac death (SCD) is an infrequent indication for cardiovascular magnetic resonance (CMR). Patients who survive an arrhythmic cardiac arrest usually receive an implantable cardioverter-defibrillator (ICD). It is important to determine a precise diagnosis in these patients as it has implications for ongoing healthcare, both for themselves and family members. It is presently unknown if CMR is useful for this purpose.

## Methods

At a large tertiary cardiac centre, electronic records from June 2006 to June 2013 were investigated to identify all patients who had ICD implantation. These records were cross referenced with all patients who had been investigated by CMR. Electronic records and medical notes were systematically reviewed to establish the details of the cardiac arrest, other cardiac investigations and etiology. Aborted SCD was defined as ventricular fibrillation (VF) or hemodynamically unstable ventricular tachycardia (VT) requiring electrical or chemical cardioversion.

## Results

152 patients had an ICD implanted and a prior CMR scan. Of these, 62 patients had the CMR to specifically investigate aborted SCD. The scan was abandoned in one because of claustrophobia and detailed records were available on 56 patients. Of these, the rhythm was primary VF in 29, torsade de pointes in 4 and haemodynamically unstable VT treated by electrical or chemical cardioversion 23. All patients underwent investigation by ECG, transthoracic echocardiography and coronary angiography. CMR was able to establish the diagnosis in 39 (70%) patients and angiography in 20 (36%). The final diagnosis was unclear after ECG, echocardiography and angiography in 22 patients. CMR revealed a diagnosis in 8 of these (2 ischaemic subendocardial scar, 2 myocarditis, 1 ARVC, 1 DCM, 1 cardiac sarcoid & 1 LV non-compaction). Of the remaining patients the diagnosis was 12 primary VF (diagnosed by the clinical team), 1 ARVC (diagnosed on non-imaging criteria) and in 1 patient the diagnosis was unclear. There were no cases when CMR yielded the diagnosis if the ECG, echocardiogram and angiography were completely normal.

## Conclusions

In this series of patients who suffered aborted SCD CMR was able to establish the diagnosis in 39/56 of all patients and 8/22 patients in whom the diagnosis was unclear after routine investigation with ECG, echocardiography and angiography. The high diagnostic yield of CMR, even in patients where routine investigations did not yield a diagnosis, supports its more widespread use in aborted SCD.

## Funding

PS is funded by a British Heart Foundation Fellowship (FS/12/88/29901).

**Figure 1:**
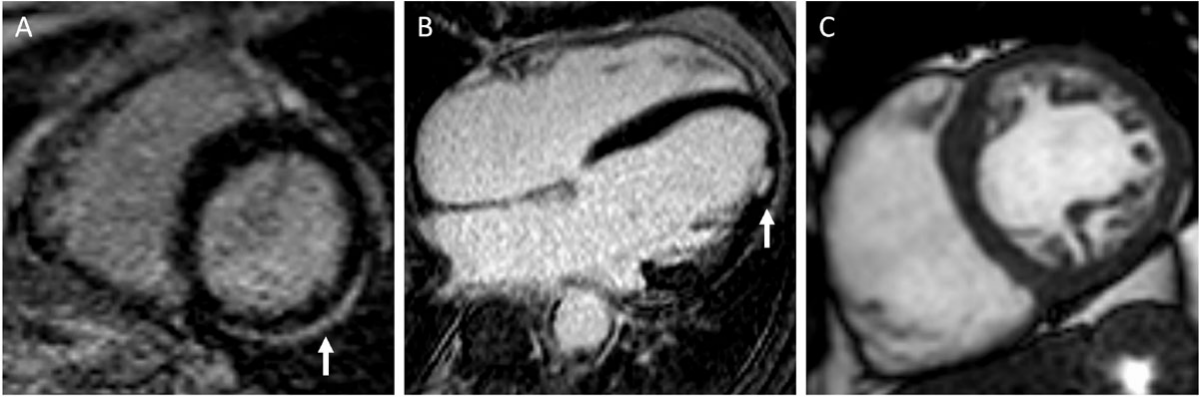
**CMR findings of 3 patients who suffered aborted SCD in whom the diagnosis was unknown after ECG, echocardiography and angiography A**. lateral subepicardial late gadolinium enhancement (myocarditis) B. lateral subendocardial late gadolinium enhancement (myocardial infarction) C. two layered myocardium with prominent trabeculation (LV non-compaction).

